# Critical Role of Neuropeptides B/W Receptor 1 Signaling in Social Behavior and Fear Memory

**DOI:** 10.1371/journal.pone.0016972

**Published:** 2011-02-24

**Authors:** Ruby Nagata-Kuroiwa, Naoki Furutani, Junko Hara, Mari Hondo, Makoto Ishii, Tomomi Abe, Michihiro Mieda, Natsuko Tsujino, Toshiyuki Motoike, Yuchio Yanagawa, Tomoyuki Kuwaki, Miyuki Yamamoto, Masashi Yanagisawa, Takeshi Sakurai

**Affiliations:** 1 Department of Molecular Neuroscience and Integrative Physiology, Faculty of Medicine, Kanazawa University, Kanazawa, Japan; 2 Exploratory Research for Advanced Technology (ERATO) Yanagisawa Orphan Receptor Project, Japan Science and Technology Agency, Tokyo, Japan; 3 Department of Neurology, Weill Medical College of Cornell University, New York Presbyterian Hospital, New York, New York, United States of America; 4 Howard Hughes Medical Institute and Department of Molecular Genetics, University of Texas Southwestern Medical Center at Dallas, Dallas, Texas, United States of America; 5 Department of Genetic and Behavioral Neuroscience, Gunma University Graduate School of Medicine, Maebashi, Japan; 6 Core Research for Evolutional Science and Technology (CREST), Japan Science and Technology Agency, Tokyo, Japan; 7 Department of Physiology, Kagoshima University Graduate School of Medical and Dental Sciences, Kagoshima, Japan; 8 Comprehensive Human Sciences, University of Tsukuba, Tsukuba, Ibaraki, Japan; University of Chicago, United States of America

## Abstract

Neuropeptide B/W receptor 1 (NPBWR1) is a G-protein coupled receptor, which was initially reported as an orphan receptor, and whose ligands were identified by this and other groups in 2002 and 2003. To examine the physiological roles of NPBWR1, we examined phenotype of *Npbwr1* ^−/−^ mice. When presented with an intruder mouse, *Npbwr1* ^−/−^ mice showed impulsive contact with the strange mice, produced more intense approaches toward them, and had longer contact and chasing time along with greater and sustained elevation of heart rate and blood pressure compared to wild type mice. *Npbwr1* ^−/−^ mice also showed increased autonomic and neuroendocrine responses to physical stress, suggesting that impairment of NPBWR1 leads to stress vulnerability. We also observed that these mice show abnormality in the contextual fear conditioning test. These data suggest that NPBWR1 plays a critical role in limbic system function and stress responses. Histological and electrophysiological studies showed that NPBWR1 acts as an inhibitory regulator on a subpopulation of GABAergic neurons in the lateral division of the CeA and terminates stress responses. These findings suggest important roles of NPBWR1 in regulating amygdala function during physical and social stress.

## Introduction

NPB and NPW were recently identified as endogenous ligands for two closely related G-protein coupled receptors, GPR7 (NPBWR1) and GPR8 (NPBWR2) [1], [2], [3]. The *NPBWR1* gene is highly conserved between the humans and rodents, while *NPBWR2* is not found in rodent genomes [4], [5]. *Npbwr1* mRNA is localized in discrete brain regions in rodents, including the hypothalamus (dorsomedial hypothalamus and suprachiasmatic nucleus), hippocampus, ventral tegmental area (VTA) and extended amygdala (CeA and bed nucleus of the stria terminalis; BST) [3], [6]. The particularly strong expression of *Npbwr1* in the CeA, together with the robust projection of NPW-containing axons to the CeA [7], suggests that this receptor might be an important modulator of the output signal from the amygdala. NPBRW1 is also abundantly expressed in other limbic regions, including the hippocampus, suggesting its roles in emotion and memory [3], [5].

In this study, we investigated potential physiological roles of NPBRW1 by studying mice with a battery of behavioral tests [8] (Table 1). While *Npbwr1* ^−/−^ mice showed normal results in many of these tests, the screening pointed to obvious abnormality of social interaction and contextual fear in these mice. Histological and electrophysiological studies revealed that NPBWR1 was expressed in GABAergic neurons in the CeA, and acted as a neuroinhibitory regulator of these neurons. These findings suggest that NPBWR1 is an important modulator of amygdala function, and that *NPBWR1* may be implicated in responses to stressful social and environmental stimuli.

**Table 1:** Summary of behavioral phenotypes of NPBWR1 knockout mice.

Behavioral test	Parameter	Results
Open field test	Anxiety	Normal time spent in center of arena
Elevated plus maze test	Anxiety	Normal time spent and number of entries in open arms
Light-dark exploration test	Anxiety	Decrease in escape latency and time spent in light box
Porsolt forced swim test	Depression, learning helplessness	Normal time spent swimming
Prepulse inhibition test	Sensory motor reactivity	Normal percentage of prepulse inhibition
Marble burying behavior test	Compulsive behavior	Normal number of marbles buried
Cued and contextual fear conditioning test	Fear and memory	Decrease in time of freezing behavior during contextual testing while normal during auditory-cued testing
Morris water maze test	Spatial memory	Normal escape latency
Resident-intruder test	Social interaction	Abnormal social interaction
Stress-induced hyperthermia	Stress response	Exaggerated hyperthermia
Daily locomotor activity	Circadian rhythm	Normal in both light/dark cycle and constant dark condition. Normal entrainment by food or light
Sleep-wake behavior (EEG/EMG)	Sleep/wake cycle	Normal in each episode duration, times spent in each state in hourly sleep/wake analysis

These observations suggest that the NPB/W system plays important roles in regulating emotion and fear memory.

## Results

### Abnormalities in Social Behaviour in *Npbwr1* ^−/−^ Mice

**Figure 1:**
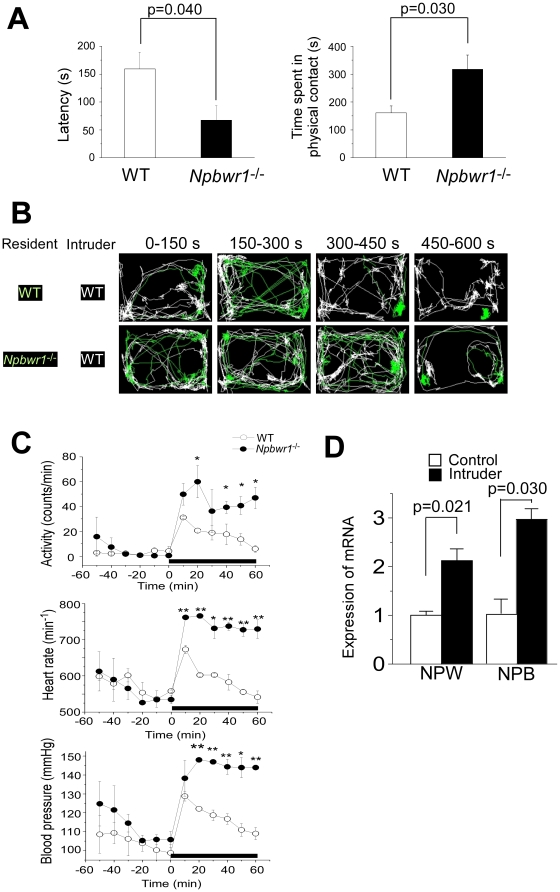
Increased impulsiveness and contact time with associated increased autonomic responses in *Npbwr1* ^−*/*−^ mice during resident-intruder test. (A) Male naive 8-week-old mice were housed individually for 4 weeks before the procedure. The behavior of mice was recorded with a CCD video camera. A randomly chosen male intruder (C57BL/6J) was used only once in each session. The intruder was introduced into the resident cage, and behavior was recorded for 10 min. A variety of social behaviors were scored including the latency to the first aggressive contact (left panel) and time spent in aggressive contact (sniffing, rattling, chasing, mounting, wrestling and fighting) (right panel). *Npbwr1* ^−*/*−^ mice showed a shorter latency time to contact with the intruder (F_1,12_ = 5.304, p = 0.040), and longer physical contact with the intruder compared with wild type mice (F_1,12_ = 6.068, p = 0.030). Data are presented as mean ± SEM (WT n = 6, KO n = 8). Also see movies S1 and S2, which show typical examples of behavior observed during this test. (B) Video tracking system shows traces of intruder (white) and resident (green) during 10 min session of resident-intruder test, showing that *Npbwr1* ^−*/*−^ mice exhibited more sustained and insistent contact and chasing behavior. Note that the trace of *Npbwr1* ^−*/*−^ mice is very similar to that of the intruder, reflecting the insistent chasing. (C) Locomotor and cardiovascular responses during resident-intruder test in radiotelemetry-implanted freely moving mice. Activity (upper panels), heart rate (HR; middle panels) and mean arterial pressure (MAP; lower panels) of resident mice (*Npbwr1* ^−*/*−^ or wild type littermates) during the time course of the resident-intruder test are shown. Intruders (male C57BL/6J mice) were put in the cages at 0 min. Horizontal solid bar indicates the presence of an intruder. Baseline values were defined as the average values of parameters obtained during 10 min immediately prior to the resident-intruder test. Data are presented as mean ± SEM (wild type; n = 4, *Npbwr1* ^−*/*−^; n = 5) (*p<0.05, **p<0.01, compared to wild-type). (D) Real time PCR analysis showed that *Neuropeptide B* (*NPB*) and *Neuropeptide W* (*NPW*) mRNAs in whole brain were upregulated after the resident-intruder test for 60 min. Each level of expression was normalized by the level of *Gapdh* mRNA (wild type; n = 45, *Npbwr1* ^−*/*−^; n = 5).

In the resident-intruder test, male *Npbwr1* ^−/−^ mice showed significantly shorter latency to initial physical contact with the intruder and a significantly longer time in contact with the intruder compared with wild type male mice (C57BL/6J) (Fig. 1A,B). The resident-intruder test also revealed that *Npbwr1* ^−/−^ mice showed characteristic behavior such as persistent chasing during the session (movies S1 and S2). They abandoned their normal caution and tendency to withdraw when confronted with a strange mouse. Instead, they impulsively approached the intruder and showed a greater frequency and duration of contact. When *Npbwr1* ^−/−^ mice were used as intruders, they again showed very fast contact with wild type resident mice and persistent chasing behavior (Fig. S1A).

Because it is well known that amygdala activation correlates with an increase in sympathetic outflow [9], we simultaneously monitored locomotor activity, heart rate (HR), and mean arterial pressure (MAP) in resident *Npbwr1* ^−/−^ and wild type littermates mice during the resident intruder paradigm, to examine the effect of social stress on these parameters. Basal activity, HR and MAP were comparable between male *Npbwr1* ^−/−^ and wild type littermates (Fig. 1C). All these parameters increased during the resident-intruder test in both *Npbwr1* ^−/−^ and control mice. However, while these parameters transiently increased and gradually returned to basal levels within 60 min in wild type controls, *Npbwr1* ^−/−^ mice showed sustained responses of activity, HR and MAP throughout the presence of the intruder. These observations suggest that *Npbwr1* ^−/−^ mice exhibit exaggerated and sustained behavioral and autonomic excitability to social stimuli. We also found that both *Npb* and *Npw* mRNAs were increased under stressful conditions induced by the resident-intruder paradigm (Fig. 1D), suggesting that this system might work as a negative feedback regulator of amygdala function. Notably, heterozygous *Npbwr1* ^+/−^ mice also showed increased locomotor activity and chasing behavior during this test, which suggests a possible gene dosage effect (Fig. S1B).

### Altered Stress Responses of *Npbwr1* ^−/−^ Mice

**Figure 2:**
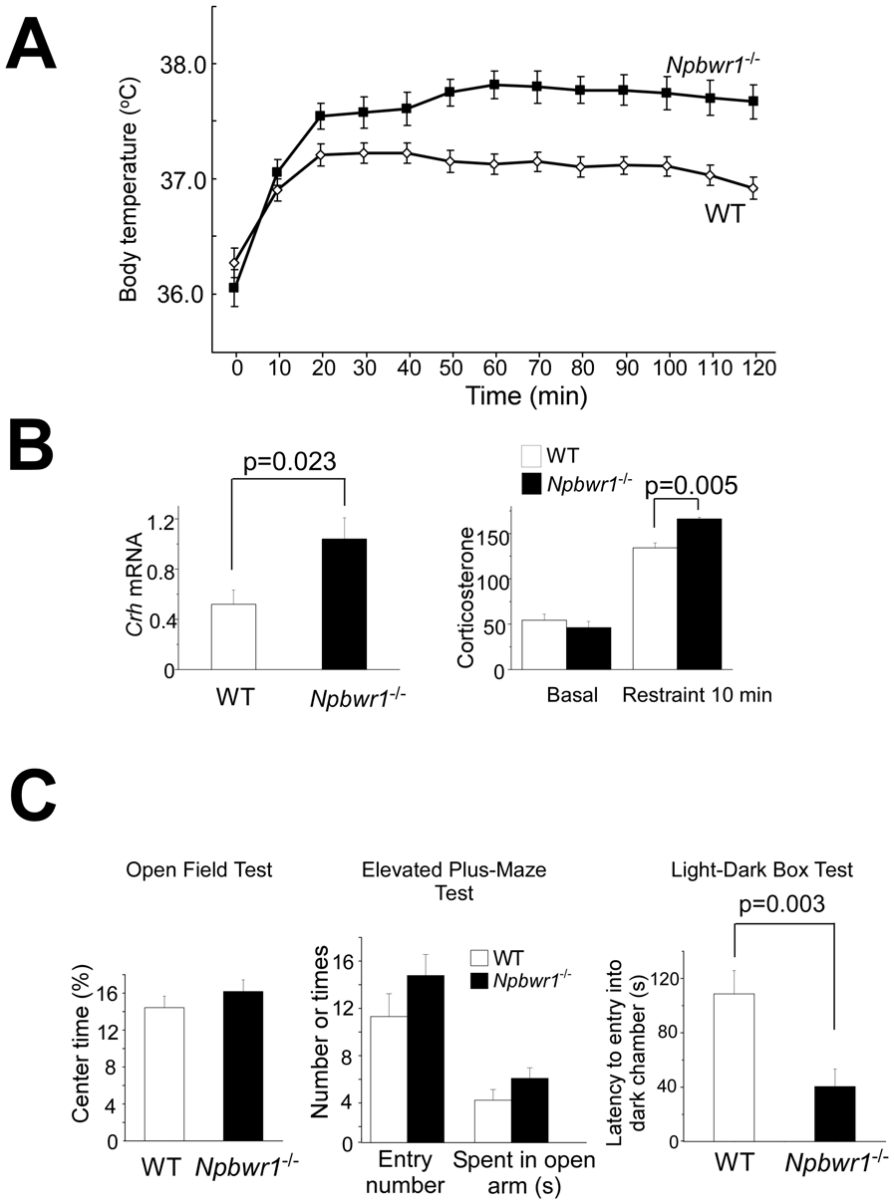
Increased autonomic, neuroendocrine and behavioral responses to physical stress in *Npbwr1* ^−*/*−^ mice. (A) *Npbwr1* ^−*/*−^ mice showed a greater increase in body temperature during repetitive handling stress (mild restriction and insertion of a probe into the rectum). (B) *Corticotropin-releasing hormone* (*Crh*) mRNA level in the hypothalamus was higher in *Npbwr1* ^−*/*−^ mice than in wild type mice (left panel) (wild type; n = 7, *Npbwr1* ^−*/*−^; n = 6, F_1,11_ = 6.928, p = 0.023). Basal serum corticosterone level in *Npbwr1* ^−*/*−^ mice was comparable to that in wild type mice (wild type; n = 12, *Npbwr1* ^−*/*−^; n = 17, F_1,27_ = 0.700, p = 0.410), but these mice showed a greater increase in corticosterone after 10 minutes of restraint stress (right panel) (wild type; n = 3, *Npbwr1* ^−*/*−^; n = 3, F_1,4_ = 30.732, p = 0.005). (C) *Npbwr1* ^−*/*−^ mice did not show overt anxiety in the basal state, but they show increased impulsiveness to environmental challenges. Left panel, open-field test. Percentage of time spent in the center was not significantly different between *Npbwr1* ^−*/*−^ mice and wild type mice (wild type; n = 23, *Npbwr1* ^−*/*−^; n = 17, F_1,38_ = 0.551, p = 0.463). Middle panel, elevated-plus maze test. Number of entries into open arms and time spent in open arms during 5 min test session were not different between genotypes (wild type; n = 20, *Npbwr1* ^−*/*−^; n = 24, F_1,42_ = 1.734, p = 0.195 and F_1,42_ = 2.089, p = 0.156, respectively). Right panel, light-dark exploration test. The total number of transitions, time spent in the light side, and latency until mice escaped to the dark side were recorded for 10 min after a single mouse was placed in the light compartment. Latency to enter the dark chamber from the light chamber is significantly shorter in *Npbwr1* ^−*/*−^ mice (wild type; n = 17, *Npbwr1* ^−*/*−^; n = 17, F_1,32_ = 10.136, p = 0.003). Data are presented as mean ± SEM.

The behavioral and autonomic abnormality of *Npbwr1* ^−/−^ mice in threatening circumstances induced by social interaction suggests that NPBWR1 plays an important role in regulation of behavioral arousal and autonomic output induced by social emotional stress in mice. To examine the roles of NPBWR1 in evoking stress responses to physical environmental challenges, we further examined the autonomic and neuroendocrine responses of *Npbwr1* ^−/−^ mice to physical stresses. We found that stress-induced hyperthermia, which is often used to examine stress responses in mice [10], [11], was significantly higher in *Npbwr1* ^−/−^ mice than in wild type mice (Fig. 2A). We also found that basal *corticotropin-releasing hormone* (*Crh*) mRNA level in the hypothalamus was higher in *Npbwr1* ^−/−^ mice than in controls (Fig. 2B). Furthermore, although the basal serum corticosterone level in *Npbwr1* ^−/−^ mice was comparable to that in control mice, possibly due to tight feedback regulation of this hormone in the basal state, it increased to a higher level after application of restraint stress for 10 min as compared with that in wild type controls (Fig. 2B). These observations further support an inhibitory role of NPBWR1 in stress-induced neuroendocrine and autonomic responses.

The increased responses to various stresses in *Npbwr1* ^−/−^ mice suggest the possibility that these mice show high anxiety. However, in the open-field test, *Npbwr1* ^−/−^ mice exhibited no abnormality in the percentage of time spent in the center of the arena (thigmotaxis), and showed no significant difference in the percentage of time spent in the open arms in the elevated-plus maze test (Fig. 2C), suggesting that the basal level of anxiety was unaltered in *Npbwr1* ^−/−^ mice. However, *Npbwr1* ^−/−^ mice showed a significantly shorter latency to first entry into the dark chamber in the light-dark exploration test (Fig. 2C). As *Npbwr1* ^−*/*−^ mice had normal thigmotaxis and a normal response in the elevated plus maze, this response to light-dark exploration might reflect increased impulsivity of *Npbwr* ^−/−^ mice to a novel physical environment rather than heightened anxiety. This is consistent with the aforementioned results of the resident-intruder test, which may be interpreted as increased impulsivity to a social challenge.

### Abnormality of Contextual Fear in *Npbwr1* ^−/−^ Mice

**Figure 3:**
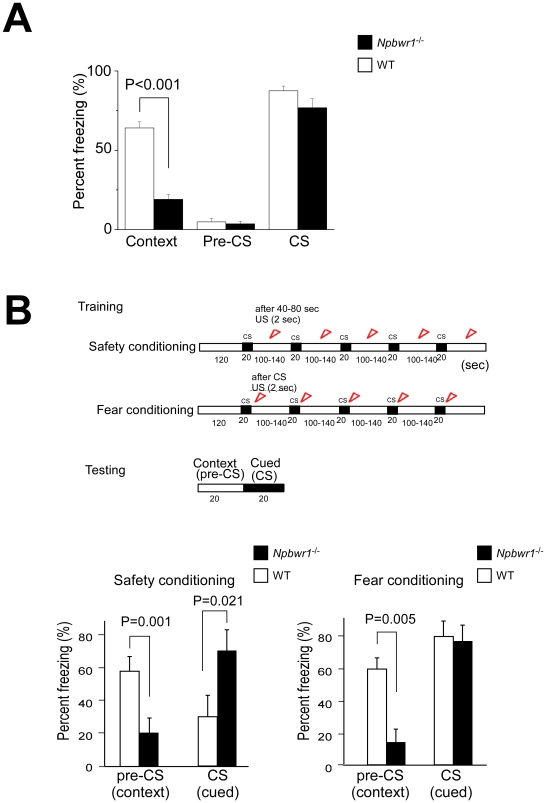
*Npbwr1* ^−*/*−^ mice showed abnormality in contextual fear conditioning. (A) Fear conditioning was performed to examine the ability of *Npbwr1* ^−*/*−^ mice to learn and remember an auditory cue or context that predicted electric shock. Bars show the mean percentage of time spent freezing (defensive tonic immobility) during 30 s observation. For contextual fear test, mice were tested in the absence of cues in the same context at 24 hr after training. For cued test, mice were tested in new cages and the auditory cue applied. Freezing behavior of mice was counted before (pre-CS) and during application of the cue. There was a significant difference in duration of freezing behavior between *Npbwr1* ^−*/*−^ mice and wild type mice during the contextual fear task (wild type; n = 13, *Npbwr1* ^−*/*−^; n = 18, F_1,29_ = 114.15, p<0.001), while no significant difference in freezing behavior was observed during auditory-cued testing under the altered context. (B) Alternative protocols for fear and safety conditioning [13]. Upper panel, schematic representation of training and testing protocols. Mice were put in the conditioning chamber for 2 min before the first stimulus. Conditioning sessions consisted of 5 CS (20 s) (interval, mean 130 s, range, 100–140 s). In safety conditioning sessions, the US was explicitly unpaired and occurred during the inter-CS interval (five US per session, separated by 20–80 s from each CS). Training sessions were conducted for three days (one session per day). In fear conditioning, the US was applied for the last two sec of the CS, which was applied at the same protocol as the safety conditioning. In the test sessions, CS was delivered at the same protocol as conditioning, and no US was delivered. Freezing times in the 20 s periods before and during CS application were scored as context and cued conditioning, respectively. Lower panels, Times spent freezing during 20 s CS and 20 s prior to CS in safety conditioning (left panel) and fear conditioning (right panel) are shown. In safety conditioning, wild type mice displayed freezing to the context, which invariably accrued with US exposure. The freezing was reduced by the arrival of the CS. However, *Npbwr1* ^−*/*−^ mice did not show fear responses to the context, and exhibited freezing to the CS. In fear conditioning, we obtained virtually the same results as those with the classical protocol (A).

The amygdala and hippocampus have long been thought to play an important role in establishment of emotional memory. We tested whether Npbwr1 plays a role in this process using classical cued and contextual fear conditioning paradigms. Mice were placed in a conditioning chamber for 2 min before being given an auditory-cued conditioned stimulus (CS), a tone, which lasted for 30 sec. The last 2 sec of the CS was paired with a mildly aversive shock unconditioned stimulus (US). For contextual fear testing, mice were tested in the absence of both CS and US in the same experimental context at 24 hr after training [12]. Although wild type control mice showed significantly increased freezing time when they were put in the same context as they were conditioned, *Npbwr1* ^−*/*−^ mice did not show increased freezing behavior during the contextual fear test. However, we did not observe a significant difference in freezing behavior between *Npbwr1* ^−*/*−^ and wild type mice during CS testing under the altered context (Fig. 3A).

To further evaluate the abnormality of fear-related memory seen in the contextual fear conditioning test, we also performed fear conditioning with a different conditioning protocol (safety conditioning [13]). In this paradigm, we used an auditory CS that was explicitly unpaired with a US (Fig. 3B). With three days of training and testing, this protocol established safety conditioning in wild type mice, and the CS signals (safety signals) significantly reduced the expression of freezing behavior to the experimental context. However, *Npbwr1* ^−*/*−^ mice showed markedly different behavioral characteristics during this test. They did not show fear responses to the experimental context after safety conditioning, and exhibited freezing behavior to the CS.

As a control experiment for the safety conditioning protocol, we performed a test with a similar protocol, but this time, the US immediately followed every occurrence of the CS. In the test session of this protocol (fear conditioning), the CS robustly increased freezing time beyond the contextual freezing level in wild type mice. In this experimental condition, *Npbwr1* ^−*/*−^ mice showed virtually the same result as in the classical protocol; they showed freezing behavior to the US, but not to the experimental context (Fig. 3B). These observations suggest that *Npbwr1* ^−*/*−^ mice have an abnormality in establishment of contextual fear memory and/or expression of fear-related behavior, although they can establish fear memory to a simple auditory cue.

### Function of NPBWR1 is Involved in Amygdala Regulation

**Figure 4:**
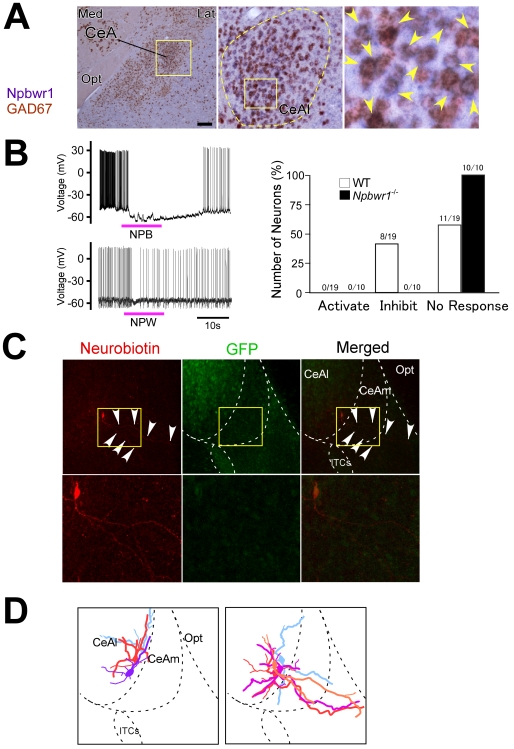
Function of NPBWR1 in regulation of CeA neurons. (A) Left panel, Dual-label In situ hybridization histochemistry showed co-localization of *Npbwr1* mRNA (blue) with *Gad67*-expressing neurons (red) in the CeAl of mice. Scale bar equals 250 *µ*m. Middle panel, higher power view of yellow rectangle region in the left panel. Right panel, high power view of yellow rectangle region in the middle panel. Opt, optic tract. (B) Left panels, typical examples of whole cell patch-clamp recording from GAD67-expressing neurons in *Gad67-gfp* brain sections, showing that bath-application of NPB (upper panel, 500 nM) or NPW (lower panel, 500 nM) potently inhibited neuronal activity. Right panel, numbers of GFP-positive neurons activated or inhibited by NPB/W application. We did not observe any effects in neurons of *Npbwr1* ^−*/*−^ mice. (C) A typical example of morphology of NPB/W-inhibited GABAergic neurons as revealed by neurobiotin injection after patch-clamp recordings. This cell resides in the medial region of the CeAl and sends long projections to outside of the amygdala. (D) Schematic drawings of axonal projections of NPW and/or NPB-inhibited neurons in the CeAl. Left panel shows three neurons depicted in different colors that send axons within the CeAl. Right panel shows four neurons that send axons outside of the CeA.

Since we found abnormality of social interaction, autonomic responses, and contextual fear conditioning, all of which are related to amygdala function, in *Npbwr1* ^−*/*−^ mice, we next explored the neuronal mechanisms by which NPBWR1 regulates the function of the amygdala, by probing the expression profile of NPBWR1 in the neural circuitry of the amygdala in mice. By double-label in situ hybridization, we found that *Npbwr1* was abundantly expressed in GAD67-positive, gamma-aminobutyric acid (GABAergic) neurons in the medial region of the lateral division of the CeA (CeAl) (Fig. 4A). *Npbwr1* mRNA was present in 34.1±5.3% (n = 3) of *Gad67*-positive neurons within the CeAl. Virtually all *Npbwr1*-positive neurons were also positive for *Gad67*, suggesting that most of the NPBWR1-positive neurons were GABAergic in the CeAl. We also observed that *Npbwr1* was expressed in *Gad67*-positive neurons in the BST, which is recognized to be an extension of the CeA [9] (Fig. S2A). These findings confirm that NPBWR1 is expressed in GABAergic neurons in the output nuclei of the extended amygdala, where NPW-immunoreactive fibers were exclusively observed in the mouse brain [5], [7] (Fig. S2B). We next examined the effect of NPB and NPW on *Gad67*-positive neurons in the CeAl by means of patch-clamp recording. Whole cell recording showed that bath application of NPB or NPW hyperpolarized and inhibited 8 out of 19 *Gad67*-positive neurons in the CeAl in slice preparations (Fig. 4B). None of the 10 neurons tested from *Npbwr1* ^−/−^ mice showed such inhibition. Neurons in the CeAl are mostly GABAergic and many of these neurons are thought to send inhibitory projections to neurons in the medial part of CeA (CeAm), the main output nucleus of the amygdala. However, a subpopulation of CeAl neurons are also known to directly project to the BST and brain stem target areas [14]. Morphological examination of NPW-inhibited cells by injecting neurobiotin after recordings showed that four out of seven NPW-inhibited neurons examined had relatively long axons that projected through the CeAm to outside the amygdala (Fig. 4C, D). These observations demonstrate that NPB/W acts on projection neurons in the CeAl. We also observed some cells with shorter axons that ended within the CeAl. Because these studies were done using slice preparations, we cannot conclude that these axons ended within the CeAl, but it is plausible that some NPBWR1-positive neurons could be GABAergic interneurons in the CeAl.

## Discussion

### Abnormality in Behavioral and Neuroendocrine Responses of *Npbwr1* ^−/−^ Mice

Our present study showed that *Npbwr1* ^−/−^ mice have abnormal behavioral and neuroendocrine responses to social and physical stresses (Figs. 1, 2). The abnormal behavior toward the intruder possibly reflects increased impulsivity to potential danger and/or inability to appropriately recognize unknown conspecifics as a threat. We hypothesized that this abnormal behavior is due to abnormal neurotransmission in the amygdala, firstly because NPBWR1 is abundantly expressed in the CeAl, the only region in which we observed NPW-ir in mouse brain [7]. Secondly, the phenotype is similar to the abnormality in humans and primates with amygdala damage. Earlier studies in nonhuman primates with bilateral amygdala lesions also showed a similar response, where the lesioned animals showed less tension-related behavior and diminished passive avoidance of potentially dangerous environmental stimuli such as a rubber snake when compared to sham-controlled animals [15], [16]. Another possibility is that *Npbwr1* ^−*/*−^ mice have a lack of personal space regulation, which may be similar to that seen in a human with bilateral amygdala lesions, who showed a lack of discomfort at close distances to others [17]. Our observations suggest that *Npbwr1* ^−*/*−^ mice have an abnormality in evoking normal caution and/or fear when confronted with strange mice.

Our findings in *Npbwr1* ^−/−^ mice of exaggerated neuroendocrine responses to various physical stresses are consistent with a neuroinhibitory effect on Npbwr1 on amygdala function, which leads to strong and sustained activation of the sympathetic division of the autonomic outflow in response to environmental stimuli.

### Abnormality of Contextual Fear in *Npbwr1* ^−/−^ Mice

Abnormality of the behavior evoked by the resident-intruder paradigm and the results of contextual fear conditioning suggest that Npbwr1 might play a role in evoking proper behavior to relatively complex polymodal environmental cues, such as contextual information for danger and social interaction. On the other hand, *Npbwr1* ^−/−^ mice can respond to simple sensory cues as shown by cued fear conditioning.

Another very interesting pair of results is the impairment of contextual fear conditioning and “inversion” of safety conditioning. In safety conditioning, mice received unpaired presentations of a tone or light CS and a shock US. Because the shock never occurred during the CS, wild type mice learned to treat the CS as a safety signal, so that fear-related behaviors, such as freezing, were inhibited during presentation of the CS. *Npbwr1* ^−/−^ mice showed “inversion” of this learning in that the safety-trained CS elicits fear rather than a reduction in fear (Fig. 3). *Npbwr1* ^−/−^ mice apparently undergo trace conditioning rather than safety conditioning in this procedure, in that they associate the CS and US across a long “trace” interval, while the control mice treat CS and US as unpaired. *Npbwr1* ^−/−^ mice seem to be more “stimulus bound”, meaning that they preferentially attend to discrete stimuli, to the exclusion of more complex, conjunctive stimuli such as contexts. This hypothesis would also explain the deficit in contextual fear conditioning, and should be confirmed in future studies.

The abnormality in contextual fear conditioning tests suggests that Npbwr1 might be involved in hippocampal function, since both the amygdala and hippocampus are necessary for establishing contextual fear memory, although we did not find any abnormality in the Morris water maze test, a hippocampus-dependent memory task (Fig. S3). We also did not find any difference in long-term potentiation in CA3 pyramidal neurons (N.F., unpublished results). Together with the strong expression of *Npbwr1* in the CeAl, and the fact that NPW-i fibers were exclusively observed in the CeAl in mice [7], this supports the notion that the abnormality of contextual fear memory in *Npbwr1* ^−/−^ mice is likely to stem from abnormal neurotransmission in the amygdala.

The LA/BLA regions of the amygdala are believed to be a principal storage site for emotional memory (US-CS association), while the CeA is implicated in output regulation. Activity of CeAl neurons could affect the level of inhibitory control in the CeAl-CeAm circuit, thereby controlling CeAm output. Since Npbwr1 is expressed in the CeA, but not LA/BLA, Npbwr1 is not likely to be involved in the storing of emotional memory. Npbwr1 might instead play an important role in evoking and controlling proper behavioral and neuroendocrine responses. *Npbwr1* ^−/−^ mice consistently showed normal freezing behavior in auditory-cued testing (Fig. 3A).

However, with the recent evidence suggesting that CeA also participates in the acquisition or expression of fear memory [14], [18], Npbwr1 may play a role in this process. Coordinated control of CeA neurons by Npbwr1 might contribute to both memory storage and proper expression of behavioral and neuroendocrine responses according to complex environmental conditions. Recent studies have shown that the CeAl neurons receive input from various regions, including the sensory thalamus, BLA and insular cortex. Therefore, this region appears to be important for coordinating and processing various sensory and internal information in establishing fear memory.

However, the involvement of the NPBWR1 in hippocampal function should not be disregarded. Indeed, the deficit in contextual fear conditioning with normal cued fear conditioning is consistent with hippocampal damage, and the neuroendocrine phenotype could be related to either hypothalamic or hippocampal abnormalities both of which express *Npbwr1* mRNA [5]. A further study would be needed to confirm the involvement of the amygdala in these abnormalities using spatially-restricted deletion of NPBWR1, knockdown or a rescue experiment using *Npbwr1* ^−/−^ mice or electrophysiological experiments using the hippocampus of mutant mice.

The results in the social interaction test are surprising as *Npbwr1* ^−/−^ mice showed an increased neuroendocrine response. In addition, the increased corticosterone response to stress suggests that these mice would be anxious, which was not obvious in the open field or elevated plus maze test, but was evident in the light-dark test. Factorial analysis of behavior in anxiety-related experiments in animals has shown that different tests reflect different underlying factors [19]. Therefore, the fact that *Npbwr1* ^−/−^ mice showed such a specific phenotype in the light/dark exploration test and not in others might simply reflect the fact that these tests measure different dimensions of anxiety-related behaviors, such as impulsivity. Abnormality of stress-induced autonomic changes may also have contributed to the finding in the resident-intruder test, because feedback of autonomic responses through the vagal nerve may contribute to overall behavioral responses in animals [20].

**Figure 5:**
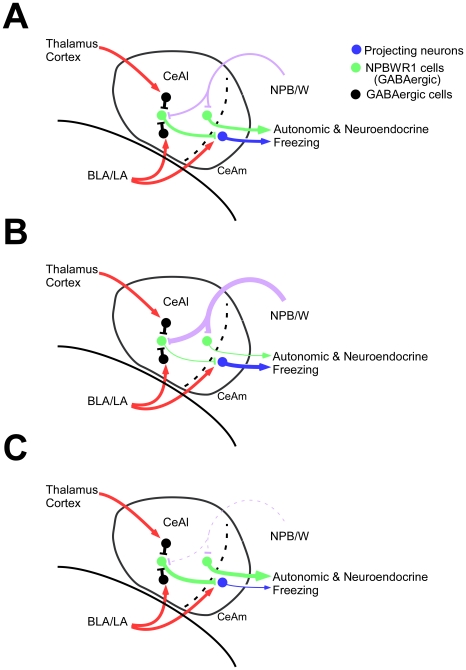
Schematic model of regulatory mechanism by which neuropeptide B/W regulates activity of amygdala neurons. (A) NPB or NPW acts on NPBWR1 expressed on projection neurons in the CeAl, which could signal to the brain stem and BST to elicit emotion-related autonomic and neuroendocrine responses. Some GABAergic interneurons in the CeAl also express Npbwr1. Therefore, NPB/W signaling could modulate amygdala function in multiple pathways. (B) When the NPB/W system is activated, some of the projection neurons in the CeAl might be inhibited, while other projection neurons might be disinhibited through inhibition of GABAergic interneurons. For example, output to autonomic/neuroendocrine pathways could be inhibited, while behavioral output might be activated. (C) NPB/W system dysfunction may result in exaggerated autonomic/neuroendocrine responses along with impaired behavioral response.

Peptidergic neuromodulation is a relatively slow and sustained process as compared to the glutamatergic and GABAergic systems. We speculate that the NPB/W system might play a role in regulating amygdala function over a relatively longer time scale. Both *Npb* and *Npw* mRNAs were increased under stressful conditions induced by the resident-intruder paradigm, suggesting that this system might work as a feedback regulator of the amygdala by inhibiting projection neurons in the CeAl (Fig. 1D). In addition, some of the GABAergic interneurons within the CeAl also expressed Npbwr1 (Fig. 4C, D). This suggests an intriguing possibility that NPB/W regulates amygdala networks by inhibiting some specific outputs while disinhibiting others, thereby helping to select proper behavioral and neuroendocrine responses [14] (Fig. 5). This model may explain why *Npbwr1* ^−/−^ mice showed decreased fear-related behavioral responses to complex contexts such as social interaction and contextual fear, but showed increased sympathetic responses to various stresses. Russel and Mehrabian classified emotions into three dimensions of factors; valence (pleasure-displeasure), arousal (autonomic response), and dominance [21]. The NPB/W system might regulate emotions in the arousal and dominance scales according to the animal's environment which contains a relatively complex context.

In human and primate genomes, there is a closely related receptor, NPBWR2, which also receives NPB and NPW as its ligands. This gene duplication during primate evolution might result in adaptation to more complex social contexts in humans and primates compared to those in rodents. Analyzing NPBWR2 function in primate social behavior is also obviously necessary to fully understand this neuropeptide system. Our ongoing study also suggests that NPW, one of the ligands for NPBWR1, plays an essential role in modulating amygdala function under stress in mice (T.M. et al., unpublished data).

In, summary, we carried out behavioral characterization of Npbwr1-deficient mice. These mice showed an intriguing pattern of behavioral abnormalities, including impaired contextual fear conditioning, impaired safety conditioning, and increased social interaction in the resident-intruder paradigm. We also observed electrophysiological changes in GABAergic neurons of the CeA, although we cannot exclude the possibility that the phenotype may be attributable to developmental loss of the gene. However, to determine the neural mechanisms and the possible developmental and/or extra-amygdalar origins of the phenotype, further investigation using spatially-restricted knockout mice and/or genetic rescue of the phenotype of these mice by expressing NPBWR1 in a region-specific manner will be clearly required in the near future. It is also clear that our findings could provide novel therapeutic targets for disorders induced by social stress, which are among the most prevalent mental health problems in the world today.

## Materials and Methods

### Animals

All experimental procedures involving animals were approved by the Animal Experiment and Use Committee of University or Kanazawa University (AP-101567), and were in accordance with NIH guidelines. *Npbwr1* ^−/−^ mice [8], in which the NPBWR1-coding region in exon 1 is disrupted by inserting a *tau-LacZ* cassette. used in the experiments were obtained from the mating of heterozygous *Npbwr1* ^+/−^ mice, which were backcrossed to wild type C57BL/6J mice for more than 10 generations. Their littermates with *Npbwr1* ^+/−^ genotype were used as wild type control. *Gad67-gfp*(*ΔNeo*) mice [22] and *Gad67-gfp*(*ΔNeo*); *Npbwr1* ^−/−^ with C57BL/6J background were used for electrophysiological and histological studies. Mice were maintained under a strict 12 hour light:dark cycle in a temperature and humidity controlled room and fed ad libitum.

### Behavioral Experiments

All behavioral experiments (Table 1) were performed during the light phase (13:00–17:00) using 8- to 14-week-old male mice. We used wild type littermates as control mice. The experimenters were blind to the genotypes until all data had been gathered and analyzed. Behavioral experiments in this study were basically performed according to protocols previously described [23]. The behavior of mice was recorded with a charge coupled device (CCD) video camera.

### Resident-Intruder Test

Male naive mice were housed individually for 4 weeks before the procedure. Isolation started at 8 weeks old. A randomly chosen intruder used only once in each session was introduced in the resident cage, and time spent in aggressive behaviors including chasing, rattling, wrestling, biting and aggressive grooming were recorded for 10 min.

### Measurement of Blood Pressure and Heart Rate in Fully Behaving Mice

Radiotelemetry implants (PA-C10; Data Sciences International; St. Paul, MN, U.S.A.) were used to monitor locomotor activity, heart rate (HR) and mean arterial pressure (MAP) in freely moving animals. The PA-C10 catheter was implanted in the left carotid artery and the transmitter body was placed in a subcutaneous pocket. All implanted mice were kept isolated, and tests were performed 7 days post-surgery to allow the mice to fully recover and their HR and MAP to return to pre-surgical levels. Before starting the test, HR, MAP and activity were recorded for 1 hour (baseline). Wild type C57BL/6J mice were used as intruders. A randomly chosen intruder was introduced in the resident cage, and HR, MAP and activity of the resident were recorded for 60 min.

### Open-Field Test

We used an open field apparatus consisting of a circular (75 cm diameter, 45 cm height) gray Plexiglas. The arena was set up under a CCD camera, and data were collected using a video tracking system, Compact VAS ver 3.0x (Muromachi Kikai, Tokyo, Japan). The floor was divided into 25 quadrants on the computer. A single mouse was placed in the center of the open field arena and its behavior was recorded for a 5-min test session. Times spent in the central quadrants and in behaviors such as rising, rearing, grooming, and voiding were evaluated as indexes of anxiety in mice. Data are presented as mean ±SEM (n = 23 and 17 for wild type and *Npbwr1* ^−/−^, respectively).

### Elevated Plus-Maze Test

We used an elevated plus-maze, constructed of Plexiglas and raised 40 cm above the floor, consisting of two opposite enclosed arms with 14 cm high opaque walls and two opposite open arms of the same size (30 cm×5 cm). Data were collected using a video tracking system compact VAS ver 3.0x. A single session lasted for 5 min. To begin a trial, the test animal was placed on the central platform facing an open arm. Anxiety levels of mice were evaluated by the percentage of entries into the open and closed arms, time spent in the open arms and distance traveled. Data are presented as mean ±SEM (n = 20 and 24 for wild type and *Npbwr1* ^−/−^, respectively).

### Light-Dark Exploration Test

The light-dark exploration test measures the tendency of mice to explore a novel environment versus the aversive properties of a brightly lit open field. The light/dark exploration test was performed using a cage (45×27×26 cm) equally divided into two (dark and light) compartments by a black partition containing a small opening. The total number of transitions, time spent in the light side, and latency until mice escaped to the dark side were recorded for 10 min after a single mouse was placed in the light compartment. Data are presented as mean ±SEM (n = 20 and 15 for wild type and *Npbwr1* ^−/−^, respectively).

### Cued and Contextual Fear Conditioning

Experiments were performed essentially as previously described [12]. On the training day, the mouse was placed in the conditioning chamber for 2 min before giving the conditioned stimuli (CS), a tone, which lasted 30 sec at 2900 Hz, 70 dB. The training was performed three times. The last 2 sec of the CS was paired with the unconditioned stimuli (US), a mild foot shock of 0.6 mA. After an additional 30 sec in the chamber, the mouse was returned to its home cage. Mice were tested 24 hr after training. Context testing was conducted in the same chamber, and freezing behavior was scored during a 30-sec testing session. Cued testing was conducted by placing the mouse in a novel environment (altered context) with the same 30 sec tone that was presented during the training day. Data are presented as mean ±SEM.

### *In Situ* Hybridization

Preparation of coronal brain sections and single *in situ* hybridization were performed according to procedures previously described [24]. For double *in situ* hybridization, each combination of two antisense riboprobes labeled with either fluorescein-UTP (*Gad1*) or digoxygenin-UTP (for *Npbwr1*) was hybridized to sections simultaneously. Following the chromogen reaction of the first color (blue) obtained with anti-digoxygenin-alkaline phosphatase (AP) Fab fragments, 5-bromo-4-chloro-3-indolyl phosphate (Roche) and nitroblue tetrazolium (Roche), sections were rinsed three times with TBS, treated twice with 0.1 M glycine pH 2.2 and 0.1% Tween 20 for 5 min, washed, and then incubated with anti-fluorescein-alkaline phosphatase (AP) Fab fragments. For the chromogen reaction of the second color (orange), 5-bromo-4-chloro-3-indolyl phosphate (Roche) and 2-[4-iodophenyl]-3-[4-nitrophenyl]-5- phenyl-tetrazolium chloride (Roche) were used.

Antisense riboprobes were synthesized from plasmids containing the coding regions of mouse *Npbwr1* (Transmembrane domain 1–5, nucleotides, 323–764) and mouse *Gad1* (NM_008077, nucleotides 281–821) cDNAs.

### Electrophysiology

*Gad67-gfp*(*ΔNeo*) mice were used for whole cell intracellular recordings. Mice were anesthetized with intraperitoneal administration of Forane (Abbott, Osaka, Japan). The mice were decapitated under deep anesthesia. The brain was isolated in ice-cold cutting solution consisting of (mM): 280 sucrose, 2 KCl, 10 HEPES, 0.5 CaCl_2_, 10 MgCl_2_, 10 glucose, pH 7.4, bubbled with 100% O_2_. Brains were cut coronally into 300-µm slices with a vibratome (VTA-1000S, Leica, Germany). Slices containing the CeAl were transferred for 1 hr to an incubation chamber at room temperature filled with physiological solution containing (mM): 140 NaCl, 2 KCl, 1 CaCl_2_, 1 MgCl_2_, 10 HEPES, 10 glucose, pH 7.4 with NaOH. The slices were transferred to a recording chamber (RC-27L, Warner Instrument Corp., CT, USA) at room temperature on a fluorescence microscope stage (BX51WI, Olympus, Tokyo, Japan). Neurons that showed EGFP fluorescence in the CeAl region were used for patch-clamp recordings. The fluorescence microscope was equipped with an infrared camera (C-3077 78, Hamamatsu Photonics, Hamamatsu, Japan) for infrared differential interference contrast (IR-DIC) imaging and a CCD camera (JK-TU53H, Olympus) for fluorescent imaging. Each image was displayed separately on a monitor (Gawin, EIZO, Tokyo, Japan). Recordings were carried out with an Axopatch 200B amplifier (Axon Instruments, Foster City, CA) using a borosilicate pipette (GC150-10, Harvard Apparatus, Holliston, MA) prepared using a micropipette puller (P-97, Sutter Instruments, Pangbourne, UK) and filled with intracellular solution (4–10 MΩ) consisting of (mM): 125 K-gluconate, 5 KCl, 1 MgCl_2_, 10 HEPES, 1.1 EGTA-Na_3_, 5 MgATP, 0.5 Na_2_GTP, 0.1% neurobiotin, pH 7.3 with KOH. Osmolarity of the solution was checked with a vapor pressure osmometer (model 5520, Wescor, Logan, UT). The osmolarities of the internal and external solutions were 280–290 and 320–330 mOsm/l, respectively. The liquid junction potential of the patch pipette and perfused extracellular solution was estimated to be −16.2 mV and was applied to the data. The recording pipette was under positive pressure while it was advanced toward individual cells in the slice. Tight seals of 0.5–1.0 GΩ were made by applying negative pressure and ZAP procedure. The membrane patch was then ruptured by suction. The series resistance during recording was 10–25 MΩ and was compensated. The reference electrode was an Ag-AgCl pellet immersed in bath solution. During recordings, cells were superfused with extracellular solution at a rate of 1.0–2.0 ml/min using a peristaltic pump (Miniplus3, Gilson, Paris, France) at room temperature.

### Statistical Analysis

Data were expressed as mean±SEM. One-way analysis of variance (ANOVA) followed by Bonfferoni method as a post-hoc test or student's t-test using Origin 6.1 software was used for statistical comparison among the various treatment groups. Differences were considered significant at p<0.05.

## Supporting Information

Figure S1 **Supplemental data for resident intruder test**. (A) Increased impulsiveness and contact time in *Npbwr1* ^−/−^ mice during resident-intruder test when intruders were wild type or *Npbwr1* ^−/−^ mice. Male naive 8-week-old wild type mice were housed individually for 4 weeks before the procedure. The behavior of mice was recorded with a CCD video camera. A randomly chosen male intruder *Npbwr1* ^−/−^ or wild type (WT) mouse (C57BL/6J) was used only once in each session. The intruder was introduced into the resident cage, and behavior was recorded for 10 min. A variety of social behaviors were scored, including the latency to the first aggressive contact (left panel) and time spent in aggressive contact (sniffing, rattling, chasing, mounting, wrestling and fighting) (right panel). *Npbwr1* ^−/−^ intruder mice showed a shorter latency time to contact with the resident compared with wildtype (wild type; n = 6, *Npbwr1* ^−*/*−^; n = 7, F_1,11_ = 5.162, p = 0.044) and longer contact time (wild type; n = 6, *Npbwr1* ^−*/*−^; n = 7, F_1,11_ = 4.643, p = 0.050). Data are presented as mean ± SEM. (B) Locomotor activity of *Npbwr1* ^−/−^ (n = 5), *Npbwr1* ^−/+^ (n = 5), and WT (*Npbwr1* ^+/+^) mice (n = 5) monitored by radiotelemetry system during resident-intruder test. Horizontal solid bar indicates the presence of an intruder. Baseline values were defined as the average of parameters obtained during 10 min immediately prior to resident-intruder test. Data are presented as mean ±SEM. * indicates p<0.05.
(TIF)

Figure S2 **CeA and BST is major effecter site for neuropeptide W.** (A) In situ hybridization histochemistry combined with GFP-immunostaining showing that *Npbwr1* mRNA is colocalized with GFP in the bed nucleus of the stria terminalis (BST) of *Gad67-gfp*(*ΔNeo*) mice. Brown staining shows GFP immunoreactivity. Blue staining shows expression of *Npbwr1* mRNA. Left panel, *Npbwr1* mRNA is colocalized with *Gad67*-expressing neurons shown by GFP-immunoreactivity in the lateral dorsal division of the BST (BSTlp). Middle panel, higher power view of region within yellow rectangle in left panel. Right panel, high power view of region within yellow rectangle in middle panel. Arrow heads show colocalization of *Npbwr1* mRNA and GFP. (B) Immunohistochemical staining demonstrating NPW-ir fibers in the CeAl in both wild type and *Npbwr1* ^−*/*−^ mice. Upper panels show sections from wild type mice and lower panels show sections from *Npbwr1* ^−*/*−^ mice. Rectangles in the left panels are shown as high power views in the right panels. Similar staining was also observed in the BST.
(TIF)

Figure S3 ***Npbwr1*** **^−/−^** **mice show normal spatial memory as measured by Morris water maze test.** *Npbwr1* ^−/−^ mice did not show a significant difference compared with wild type mice, even in the retention phase, transfer phase, and probe trial (WT n = 15, KO n = 13). Data are presented as mean ±SEM. The apparatus consisted of a circular pool (40 cm high×120 cm diameter) filled with water maintained at 25°C and made opaque by addition of nontoxic white paint. Visual cues were placed around the pool. The escape platform, made of Plexiglas, was positioned such that its top surface was 1 cm below the surface of the water. Data were collected using a video tracking system Compact VAS ver 3.0x (Muromachi Kikai, Tokyo, Japan). The experiment was conducted in four phases. The first phase consisted of 2 days with the platform visible. This tested the ability of the animal to successfully conduct the task, particularly its visual ability to see the room cues and its motor ability to swim in the pool. For each trial during the training phase, the platform was hidden in the same quadrant and the test mouse was placed in the pool from different quadrants, and its time to reach the platform was recorded. At the end of training, a probe trial was performed where each mouse was tested to see if it could identify its spatial location. For the probe trial, the hidden platform was removed from the pool, and then the mouse was placed in the pool as before. The time each mouse spent in the quadrant that formerly contained the platform for 60 sec was recorded. Five days after the training phase, a retention phase was conducted to test for long-term memory. Finally, after the retention phase, the hidden platform was placed in the opposite quadrant and each mouse was retrained to the new platform location (transfer phase). This trial tested reversal learning in the mice. For each phase, four trials per day were given to each mouse. Each trial lasted a maximum of 90 sec (except the probe trial) with a 15 min interval between trials.
(TIF)

Movie S1 **Typical behavior of wild type C57Bl/6J mouse during resident-intruder test.** The wild type resident mouse maintained an appropriate personal space, exercising caution towards the intruder.
(MPG)

Movie S2 **Typical behavior of** ***Npbwr1*** **^−/−^** **mouse during resident-intruder test.** The resident *Npbwr1* ^−/−^ mouse showed insistent chasing of the intruder. They abandoned their normal caution and tendency to withdraw when confronted with a strange mouse. Instead, they impulsively approached the intruder and showed a greater frequency and duration of contact.
(MPG)
