# Supplementary figures and images for: Critical Role of Neuropeptides B/W Receptor 1 Signaling in Social Behavior and Fear Memory
Source: PLoS One. 2011 Feb 24;6(2):e16972. doi: 10.1371/journal.pone.0016972 (PMC3044739; doi:10.1371/journal.pone.0016972)

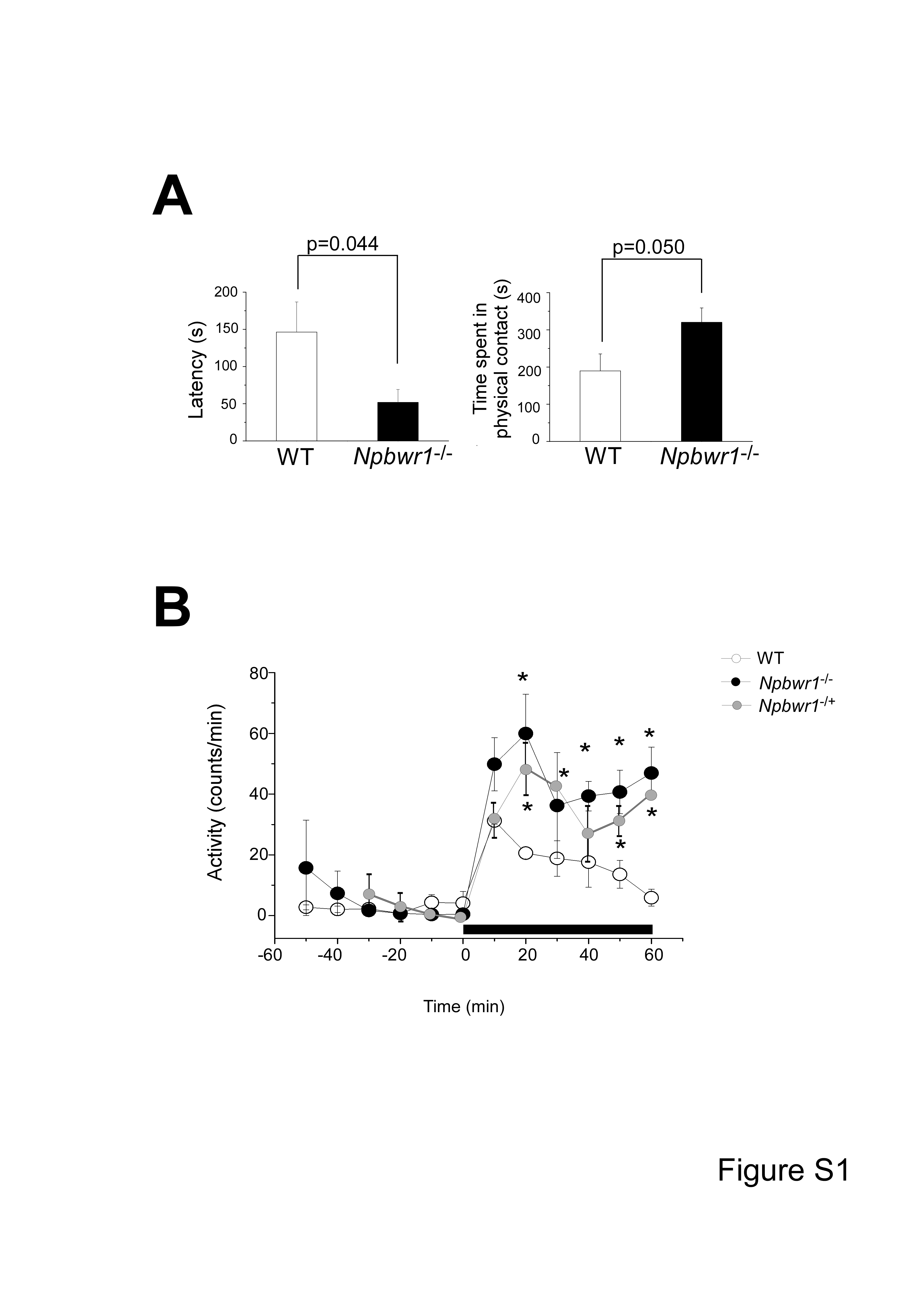

Supplement: Figure S1 — Supplemental data for resident intruder test. (A) Increased impulsiveness and contact time in Npbwr1 −/− mice during resident-intruder test when intruders were wild type or Npbwr1 −/− mice. Male naive 8-week-old wild type mice were housed individually for 4 weeks before the procedure. The behavior of mice was recorded with a CCD video camera. A randomly chosen male intruder Npbwr1 −/− or wild type (WT) mouse (C57BL/6J) was used only once in each session. The intruder was introduced into the resident cage, and behavior was recorded for 10 min. A variety of social behaviors were scored, including the latency to the first aggressive contact (left panel) and time spent in aggressive contact (sniffing, rattling, chasing, mounting, wrestling and fighting) (right panel). Npbwr1 −/− intruder mice showed a shorter latency time to contact with the resident compared with wildtype (wild type; n = 6, Npbwr1 −/−; n = 7, F1,11 = 5.162, p = 0.044) and longer contact time (wild type; n = 6, Npbwr1 −/−; n = 7, F1,11 = 4.643, p = 0.050). Data are presented as mean ± SEM. (B) Locomotor activity of Npbwr1 −/− (n = 5), Npbwr1 −/+ (n = 5), and WT (Npbwr1 +/+) mice (n = 5) monitored by radiotelemetry system during resident-intruder test. Horizontal solid bar indicates the presence of an intruder. Baseline values were defined as the average of parameters obtained during 10 min immediately prior to resident-intruder test. Data are presented as mean ±SEM. * indicates p<0.05. (TIF) [file pone.0016972.s001.tif]

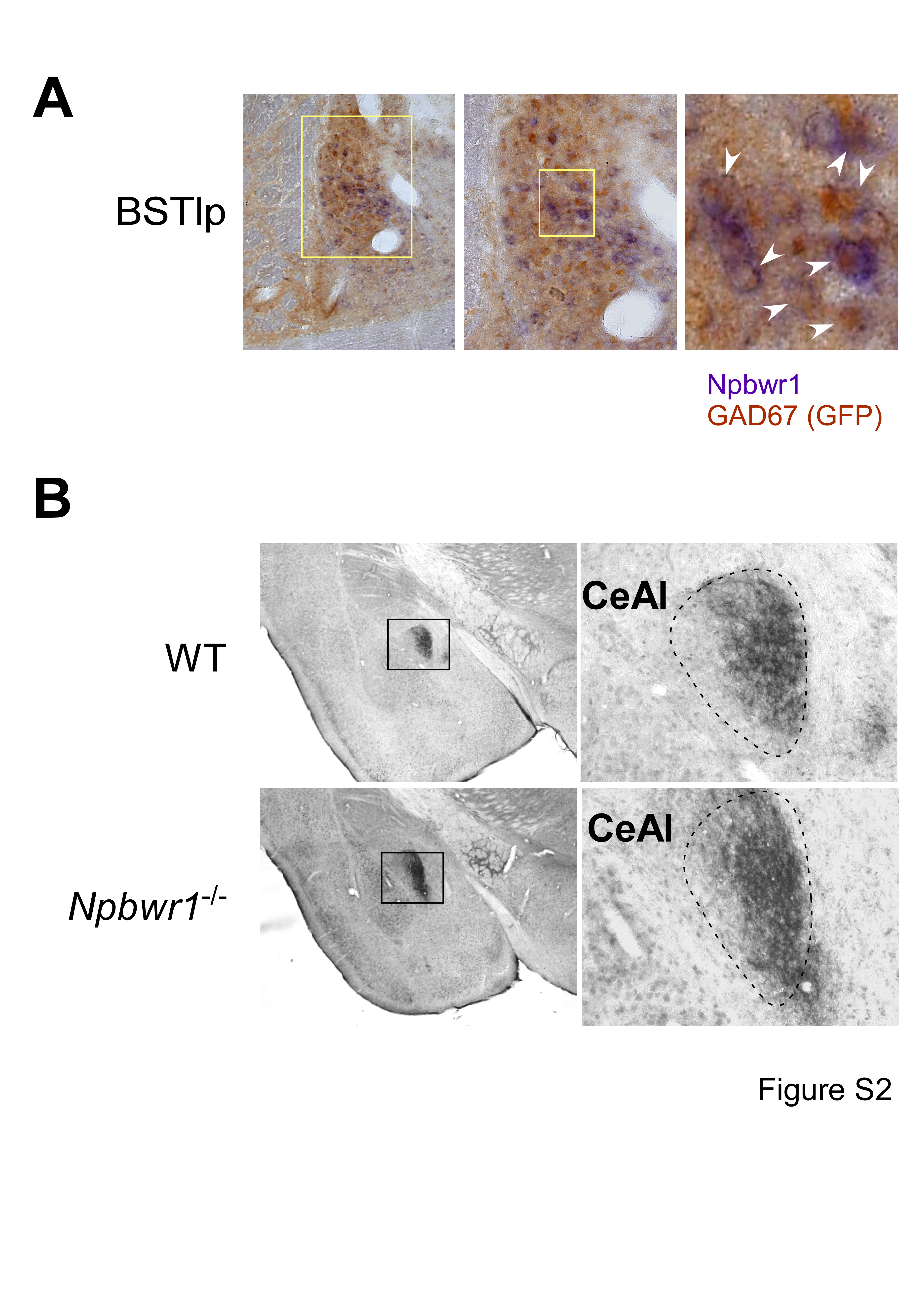

Supplement: Figure S2 — CeA and BST is major effecter site for neuropeptide W. (A) In situ hybridization histochemistry combined with GFP-immunostaining showing that Npbwr1 mRNA is colocalized with GFP in the bed nucleus of the stria terminalis (BST) of Gad67-gfp(ΔNeo) mice. Brown staining shows GFP immunoreactivity. Blue staining shows expression of Npbwr1 mRNA. Left panel, Npbwr1 mRNA is colocalized with Gad67-expressing neurons shown by GFP-immunoreactivity in the lateral dorsal division of the BST (BSTlp). Middle panel, higher power view of region within yellow rectangle in left panel. Right panel, high power view of region within yellow rectangle in middle panel. Arrow heads show colocalization of Npbwr1 mRNA and GFP. (B) Immunohistochemical staining demonstrating NPW-ir fibers in the CeAl in both wild type and Npbwr1 −/− mice. Upper panels show sections from wild type mice and lower panels show sections from Npbwr1 −/− mice. Rectangles in the left panels are shown as high power views in the right panels. Similar staining was also observed in the BST. (TIF) [file pone.0016972.s002.tif]

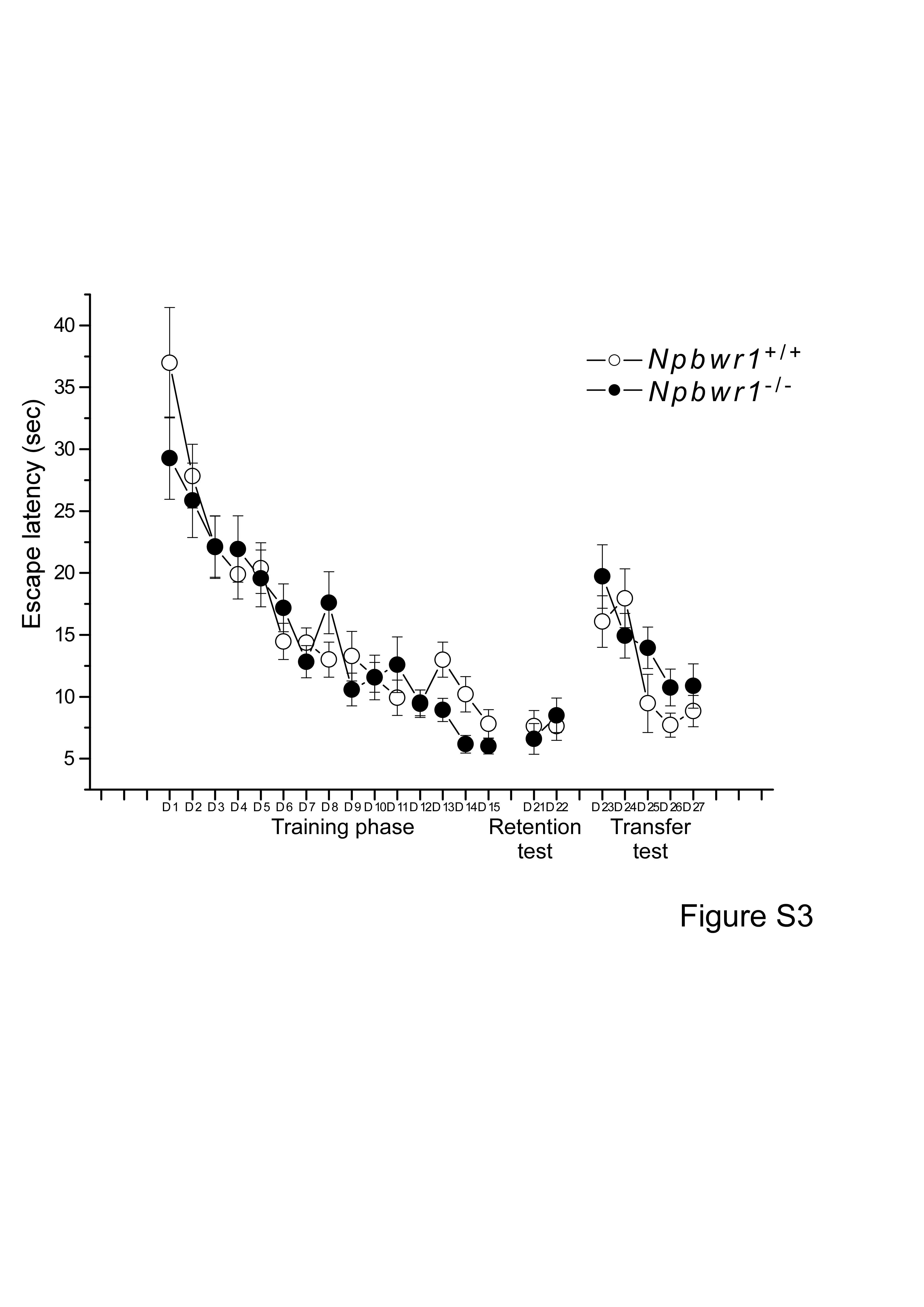

Supplement: Figure S3 — Npbwr1 −/− mice show normal spatial memory as measured by Morris water maze test. Npbwr1 −/− mice did not show a significant difference compared with wild type mice, even in the retention phase, transfer phase, and probe trial (WT n = 15, KO n = 13). Data are presented as mean ±SEM. The apparatus consisted of a circular pool (40 cm high×120 cm diameter) filled with water maintained at 25°C and made opaque by addition of nontoxic white paint. Visual cues were placed around the pool. The escape platform, made of Plexiglas, was positioned such that its top surface was 1 cm below the surface of the water. Data were collected using a video tracking system Compact VAS ver 3.0x (Muromachi Kikai, Tokyo, Japan). The experiment was conducted in four phases. The first phase consisted of 2 days with the platform visible. This tested the ability of the animal to successfully conduct the task, particularly its visual ability to see the room cues and its motor ability to swim in the pool. For each trial during the training phase, the platform was hidden in the same quadrant and the test mouse was placed in the pool from different quadrants, and its time to reach the platform was recorded. At the end of training, a probe trial was performed where each mouse was tested to see if it could identify its spatial location. For the probe trial, the hidden platform was removed from the pool, and then the mouse was placed in the pool as before. The time each mouse spent in the quadrant that formerly contained the platform for 60 sec was recorded. Five days after the training phase, a retention phase was conducted to test for long-term memory. Finally, after the retention phase, the hidden platform was placed in the opposite quadrant and each mouse was retrained to the new platform location (transfer phase). This trial tested reversal learning in the mice. For each phase, four trials per day were given to each mouse. Each trial lasted a maximum of 90 sec (except the probe trial) with a 15 mi [file pone.0016972.s003.tif]
